# Tea Polysaccharides Ameliorates Non-Alcoholic Fatty Liver Disease in Mice via Regulating Macrophages Polarization by Gut Microbial Metabolites

**DOI:** 10.3390/cimb48030338

**Published:** 2026-03-23

**Authors:** Daixin Liu, Ang Li, Ping Li

**Affiliations:** School of Life Science and Technology, Tongji University, 1239 Siping Road, Shanghai 200092, China; 2180211@tongji.edu.cn

**Keywords:** tea polysaccharides, non-alcoholic fatty liver disease, macrophage polarization, gut microbial metabolites

## Abstract

Non-alcoholic fatty liver disease (NAFLD) is the most common chronic liver disease and a global public health concern, for which there is currently no effective method to inhibit its progression. The pathogenesis of NAFLD is related to hepatic lipid metabolism disorders and liver inflammation. Previous studies have shown that tea polysaccharides (TPS) have the ability to regulate lipid metabolism and control inflammation. This study aimed to observe the effect of TPS on ameliorating NAFLD in a mouse model and to reveal its underlying mechanisms. In the current study, male C57BL/6J mice were fed a high-fat diet and administered 100 mg/kg TPS daily by gavage for 14 weeks. Then, liver injury indicators and macrophage polarization markers were detected. The results revealed that TPS could significantly ameliorate the progression of NAFLD and decrease liver injury indicators. Moreover, we found that treatment of NAFLD model mice with TPS could skew liver macrophages polarization from M1 to M2 type, which inhibited pro-inflammatory cytokines production and liver inflammation. Mechanistically, TPS cannot directly regulate the polarization of liver macrophages, but instead promotes the production of butyric acid by gut microbiota, which in turn regulates macrophage polarization. These findings suggest that TPS ameliorates NAFLD-associated inflammation by modulating the gut–liver axis and promoting M2 macrophage polarization, laying the foundation for the potential of TPS in the development of health foods for NAFLD.

## 1. Introduction

Non-alcoholic fatty liver disease (NAFLD) is a liver disease associated with metabolic disorders. Given the significant role of metabolic dysfunction in this disease, many scholars have suggested renaming it as metabolic dysfunction-associated fatty liver disease (MAFLD) [1]. The global incidence rate of NAFLD is approximately 25% [2] and is a public health concern [3]. The pathogenesis of NAFLD is characterized by a “multiple-hit” process, where insulin resistance-driven lipolysis in adipose tissue leads to an excessive flux of free fatty acids to the liver, subsequently triggering endoplasmic reticulum stress, oxidative damage, and a robust inflammatory cascade [4,5,6,7].

Recent research has found that liver macrophages play an important role in regulating liver lipid metabolism, promoting inflammation, and mediating fibrosis during the NAFLD progression [8,9]. The expansion of liver macrophages correlates with insulin resistance and oxidative stress [10]. These cells encompass both tissue-resident Kupffer cells and monocyte-derived macrophages that infiltrate from peripheral blood [9]. Transcriptomic analysis of hepatic macrophages in mice after 15–20 weeks of high-fat diet feeding demonstrated that, under conditions of obesity and steatosis, inflammatory markers are selectively expressed in monocyte-derived macrophages rather than in resident Kupffer cells [9]. Under the different environments, the macrophages polarize toward different phenotypes, namely M1 and M2 macrophages [11]. It is generally believed that M1 macrophages produce a large amount of pro-inflammatory cytokines that promote inflammation and cause liver damage, while M2 type macrophages secrete cytokines that promote the production of angiogenic factors and are involved in tissue repair and amelioration of insulin resistance [12]. Recently, some studies showed that a population of CCR2-dependent, lipid-associated macrophages, which exhibit an M1 phenotype and display impaired functions and an increased pro-inflammatory phenotype during the progression of NASH compared with non-lipid-associated macrophages [13]. Furthermore, a high-fat diet can increase plasma endotoxin levels and alter gut microbial metabolites by changing the gut microbiota, promoting overgrowth of small intestinal bacteria, and increasing intestinal permeability. Endotoxin (LPS) promotes the polarization of liver macrophages towards M1 type through LPS-Toll-like receptor 4 (TLR4) signaling pathway, thereby facilitating the progression of NAFLD [14]. Therefore, identifying effective components that can suppress the M1 polarization of hepatic macrophages will be crucial for alleviating the progression of NAFLD.

In recent years, oolong tea and black tea have attracted considerable interest for their potential protective effect on NAFLD development. Previous studies have demonstrated that oolong tea and dark tea extracts can alleviate hepatic steatosis and inflammation and reduce oxidative stress by regulating gut microbial metabolites generated from lipids such as short-chain fatty acids (SCFAs), which can modulate liver macrophage polarization via gut–liver axis [15]. For example, propionate and butyrate suppress the production of LPS-induced cytokines, including IL-12p40 and IL-6, in human mature dendritic cells [16].

Tea polysaccharides (TPS) are abundant in black tea and oolong tea and have demonstrated significant potential in modulating lipid metabolism and systemic inflammation [17,18]. In the DSS (dextran sulfate sodium)-induced mouse colitis model, TPS significantly reduced colon tissue damage and decreased disease activity through regulating intestinal microbiota, increasing intestinal mucus secretion and repairing intestinal barrier function [19]. Furthermore, TPS can alleviate inflammation in adipose tissue by regulating macrophage polarization (shifting from M1 to M2), which ameliorates insulin resistance and hyperglycemia. Therefore, we speculate that TPS, as effective components in black tea and oolong tea, can mitigate the progression of NAFLD and alleviate the liver damage by altering microbiota composition and gut metabolite profiles, thereby shifting hepatic macrophage polarization toward an anti-inflammatory phenotype.

In this study, mice fed to a high-fat diet were given 100 mg/kg TPS daily by gavage for 14 weeks to observe the preventive effect of TPS on NAFLD development. Meanwhile, we will investigate the effect of TPS on the polarization of liver macrophages in high-fat diet mice and explore the mechanisms through analysis of inflammation factors and intestinal metabolites. These findings from this study will lay the foundation for the application of TPS in the development of health foods to inhibit NAFLD.

## 2. Materials and Methods

### 2.1. Animals and Chemicals Used in the Study

Male C57BL/6J mice aged 6–8 weeks (20 ± 2 g) were sourced from Shanghai Laboratory Animal Co., Ltd. (Shanghai, China) and maintained under specific pathogen-free conditions at Tongji University. The housing environment was controlled at 22–24 °C, 50–60% relative humidity, with a 12 h light/dark cycle. All animal procedures received approval from the Institutional Animal Care and Use Committee of Tongji University (Approval No. 2025-DW-272).

Lipopolysaccharides (LPS) were obtained from Sigma-Aldrich (St. Louis, MO, USA). Acetate, propionate, isobutyrate, and butyrate were supplied by Sinopharm Chemical Reagent Co., Ltd. (Shanghai, China). Cell culture reagents, including high-glucose DMEM, RPMI 1640 medium, fetal bovine serum, trypsin, penicillin, and streptomycin, were purchased from Thermo Fisher Scientific (Waltham, MA, USA). The RT-PCR kit was acquired from Takara Bio Inc (Kusatsu, Shiga, Japan).

### 2.2. Extraction and Purification of TPS

Black tea was crushed and sieved through a 20-mesh sieve. The tea powder was then weighed and soaked overnight in an 80% ethanol solution at a ratio of 1:8 g/mL. After filtration with double-layer gauze, the tea is transferred to a fume hood to ensure complete evaporation of ethanol. Next, the tea was extracted with 500 mL of 95 °C distilled water at a ratio of 1:10 (g/mL) for 4 h. During the extraction process, the mixture was stirred several times and then filtered through double-layer gauze. The filter residue was re-extracted three times with hot water. The combined filtrate was vacuum-concentrated to one-fifth of its original volume, followed by the addition of anhydrous ethanol to achieve an 80% (*v*/*v*) ethanol concentration. The mixture was then transferred to a refrigerator at 4 °C for 24 h, followed by centrifugation to discard the supernatant. The precipitate was washed twice with acetone and anhydrous ethanol, and then freeze-dried to obtain crude polysaccharides. Next, the dried crude polysaccharides were dissolved in distilled water, and chloroform was added at a weight ratio of 1:10 to remove proteins using the Sevag method. The chloroform-n-butanol mixture was vigorously shaken for 10 min, followed by centrifugation to remove the denatured protein layer. This deproteinization process was repeated three times. Then, the collected solution was dialyzed with distilled water, followed by vacuum concentration and freeze-drying to obtain refined TPS. The purity and quality of the refined TPS were detected by phenol sulfuric acid assay, Bradford assay and HPLC (Figure S1) [20].

### 2.3. HPLC Analysis

High-performance liquid chromatography (HPLC) was employed to characterize the TPS. In brief, 2 g of TPS was treated with 10 mL of 3 M trifluoroacetic acid to release constituent monosaccharides. The hydrolysates were derivatized with 1-phenyl-3-methyl-5-pyrazolone (PMP) by adding 50 μL of 0.5 M PMP in methanol and 50 μL of 0.3 M NaOH, followed by vortex mixing and incubation at 60 °C for 1 h. The reaction was terminated by neutralization with 50 μL of 0.3 M HCl. The derivatized samples were extracted three times with 1 mL of chloroform, and the aqueous phase was passed through a 0.45 μm membrane filter prior to chromatographic analysis at 250 nm. Separation was performed using an Agilent Technologies 2004 system equipped with a quaternary pump, UV–vis detector, and autosampler (Santa Clara, CA, USA). A C18 column (4.6 × 250 mm, 5 μm; Agilent, Santa Clara, CA, USA) was used at 35 °C with a flow rate of 1.0 mL/min. The mobile phase consisted of water (A) and acetonitrile (B), with a linear gradient from 90% to 86% B over 40 min. The injection volume was set at 20 μL.

### 2.4. Mice Grouping and Drug Administration Protocol

A 14-week preventive experiment was designed to explore the preventive effect of TPS on NAFLD. After a one-week acclimatization period, the C57BL/6J mice were randomly assigned to the following four groups: a control group maintained on a regular diet (CD, Suzhou Double Lion Laboratory Animal Feed Technology Co., Ltd. Suzhou, China), a high-fat diet model group (HFD, 60% fat, D12450J, Research Diet, New Brunswick, NJ, USA), a CD group treated with TPS (CD+TPS) and an HFD group treated with TPS (HFD+TPS). Mice in the TPS treatment groups (CD+TPS or HFD+TPS) were administered an aqueous TPS extract (90% purity) daily at a dose of 100 mg/kg/day via oral gavage. TPS was dissolved in sterilized water. Every group contains eight mice. Throughout the study, all animals had free access to purified drinking water. The liver, colon and blood were collected at the end of the experiments after euthanasia. The blood was centrifuged at 3000× *g* to get serum, and the remaining tissues were preserved at −80 °C for subsequent analysis. For the antibiotic interference experiment, a subset of HFD and HFD+TPS mice received an antibiotic cocktail (1.0 mg/mL ampicillin, 1.0 mg/mL metronidazole, 1.0 mg/mL neomycin, and 0.5 mg/mL vancomycin) in their drinking water until euthanasia. To achieve macrophage depletion, clodronate-loaded liposomes (Clodronate Liposomes, FormuMax, Sunnyvale, CA, USA) were administered intraperitoneally at a dose of approximately 1 mg in 200 µL, starting from week 3 and repeated twice weekly until euthanasia. The dosage was determined according to the manufacturer’s guidelines.

### 2.5. Liver Histopathology and Oil Red O Staining

The histopathological assessment of liver tissues was conducted using hematoxylin and eosin (H&E) staining and Oil red O staining. These procedures were adapted from established methodologies with minor adjustments [21,22]. For H&E staining, liver samples were fixed in 4% paraformaldehyde and subsequently embedded in paraffin. Then, the blocks were cut into 4–5 μm slices and then stained with H&E. For Oil red O staining, tissues were frozen in optimal cutting temperature compound (OCT 4583, Beyotime Bio, Shanghai, China) and were cut into 5–6 μm slices. The sections were fixed with 10% neutral buffered formalin for 10–15 min, followed by incubation in a filtered Oil Red O working solution for 10–15 min to stain neutral lipids. The sections were then differentiated briefly in 60% isopropanol to remove background staining, followed by rinsing with distilled water. For immunofluorescence staining, tissue sections were first blocked with 5% BSA at room temperature for one hour. They were then incubated overnight at 4 °C with primary antibodies against M1/M2 markers (CD206-FITC and iNOS-PE, each diluted 1:1000). Following primary antibody incubation, the sections were washed three times with PBS, 8 min per wash. After removing residual PBS, the sections were treated with an appropriate secondary antibody for 50 min, followed by another three washing steps. Imaging was subsequently performed using a ZEISS Axio Imager upright microscope (Carl Zeiss AG, Oberkochen, Germany).

### 2.6. NAFLD Activity Score (NAS) Calculation

NAS = Steatosis score + Lobular inflammation score + Hepatocyte Ballooning score. 1. Steatosis score (Fatty Change): evaluating the percentage of hepatocytes containing fat vacuoles. Score 0: <5%; score 1: 5–33%; score 2: >33–66%; score 3: >66%. 2. Lobular inflammation score: evaluating the number of inflammatory foci per 200× microscopic field. Score 0: no foci; score 1: <2 foci per 200× field; score 2: 2–4 foci per 200× field; score 3: >4 foci per 200× field. 3. Hepatocyte ballooning score: evaluating the presence of injured, swollen hepatocytes, often with rarefied cytoplasm. Score 0: none; score 1: few, obvious balloon cells; score 2: many/prominent balloon cells.

### 2.7. Detection of Biochemical and Oxidative Stress Markers of Liver and Serum

Liver tissue was homogenized in cold saline (1:10, *w*/*v*) and centrifuged twice (3000 rpm/min, 10 min) to obtain the supernatant. This supernatant was then analyzed for oxidative stress markers in the liver, including GSH-Px and SOD, with corresponding commercial kits (Nanjing Jiancheng Bioengineering Institute, Nanjing, China) in accordance with the manufacturer’s instructions. Serum ALT (Cat. C009-2-1), AST (Cat. C010-2-1) and MDA (Cat. A003-1-2) were detected with corresponding commercial kits (Nanjing Jiancheng Bioengineering Institute, China) in accordance with the manufacturer’s instructions. Additionally, an automatic biochemical analyzer (BS-200, Mindray, Shenzhen, China) was employed to determine serum profiles, including TG (Cat. 141721006), TC (Cat. 141521008), HDL-C (Cat. 142021007), and LDL-C (Cat. 142021009) in accordance with the manufacturer’s instructions.

### 2.8. Macrophage Sorting and Culture

Mice were anesthetized and the ventricles were perfused with sterile 4 °C PBS. When the liver turned white, it was cut with sterile scissors and placed in sterile 4 °C PBS. The liver tissue was ground and filtered to obtain a single-cell suspension, followed by washing the cells with PBS twice. The cells were then collected, and Percoll (Cat.P8370, Solarbio, Beijing, China) gradient centrifugation was used to perform initial sorting of macrophages; after the initial sorting, F4/80 flow cytometry antibodies Cat.11-4801-82, eBioscience, San Diego, CA, USA) were used to label macrophages. Flow cytometry sorting was performed to isolate F4/80-labeled macrophages at 98% purity. The sorted macrophages were placed in a 6-well plate (5 × 10^5^/well) and treated with LPS (50 ng/mL), LPS (50 ng/mL) + TPS (1 mM) or LPS (50 ng/mL) + butyric acid (2 mM). After 24 h, the macrophages were collected, and qRT-PCR was performed to detect M1/M2-related genes.

### 2.9. ELISA

After blood collection, the tube was allowed to stand for 30 min, placed on ice for 20 min, and then centrifuged at 14,000× *g* and 4 °C for 10 min. The serum was then collected for the detection of LPS concentration by ELISA (Cat. KBR-hlk1718, Coibo Biotec Inc., Shanghai, China). This assay was performed according to the manufacturer’s protocols and read at 450 nm with a microplate reader (Bio-Rad Laboratories, Inc., Hercules, CA, USA). The level of LPS was calculated based on a standard curve.

### 2.10. Quantitative Real-Time PCR (qRT-PCR) Assay

RNA was isolated with Trizol reagent (Takara, Dalian, China), followed by cDNA synthesis using a commercial reverse transcription kit (Takara, Dalian, China) according to the manufacturer’s protocol. Quantitative real-time PCR (qRT-PCR) was conducted on a Light Cycler 96 system (Roche, Indianapolis, IN, USA) with SYBR Green mix (Takara), under the following cycling conditions: 95 °C for 20 s, 56 °C for 30 s, and 72 °C for 40 s, repeated for 35 cycles. Relative gene expression levels were determined using the 2^−ΔΔCT^ method, with GAPDH serving as the internal control for the analysis of mouse IL-1β, IL-10, iNOS, TNF-α, and CD206. Primers sequences were shown in Table 1.

### 2.11. Short-Chain Fatty Acid Analysis

Gas chromatography was employed to quantify the levels of acetate, propionate, isobutyrate and butyrate to get the standard curve. For sample processing, 300 mg of mouse colon tissue was mixed with 25% ortho-phosphoric acid (4.36 mol/L), 300 μL of 4-methylvaleric acid (23.83 μmol/mL; Sigma-Aldrich, St. Louis, MO, USA) serving as the internal standard, and double-distilled water. The mixtures were subsequently sonicated, vigorously vortexed, and centrifuged (20,000× *g*, 4 °C, 20 min). Then the harvested supernatant was filtered through a 0.22 μm sterile membrane and carefully transferred to a sample vial for GC–MS analysis. The clarified supernatants obtained were injected into a GC-2010 Plus Capillary Gas Chromatograph (Shimadzu Corp., Kyoto, Japan) for SCFAs analysis. The GC system, equipped with a 30 m × 0.53 mm × 0.5 μm capillary column (Trace TR Wax, Thermo Fisher Scientific) and the Thermo Q Exactive mass spectrometer with the spray voltage of 3.5 kV and 2.5 kV in positive and negative modes, respectively. Helium was used as the carrier gas. A 1 µL sample was introduced via an autosampler (AOC-20s/AOC-20i, Shimadzu, Kyoto, Japan). Finally, SCFAs were quantified by the standard curves of acetic acid, propionic acid and butyric acid.

### 2.12. Statistical Analysis

All statistical analyses and graphical presentations were conducted with GraphPad Prism (version 8.01, San Diego, CA, USA). Data from two-group comparisons are expressed as mean ± standard deviation ($\bar{x}$ ± s) based on normality test outcomes. For normally distributed and homoscedastic datasets, Student’s *t*-test was applied. For multi-group comparisons, one-way ANOVA was used. A *p*-value below 0.05 was deemed statistically significant.

## 3. Results

### 3.1. Effect of TPS on High-Fat Diet-Induced NAFLD in Mice

**Figure 1:**
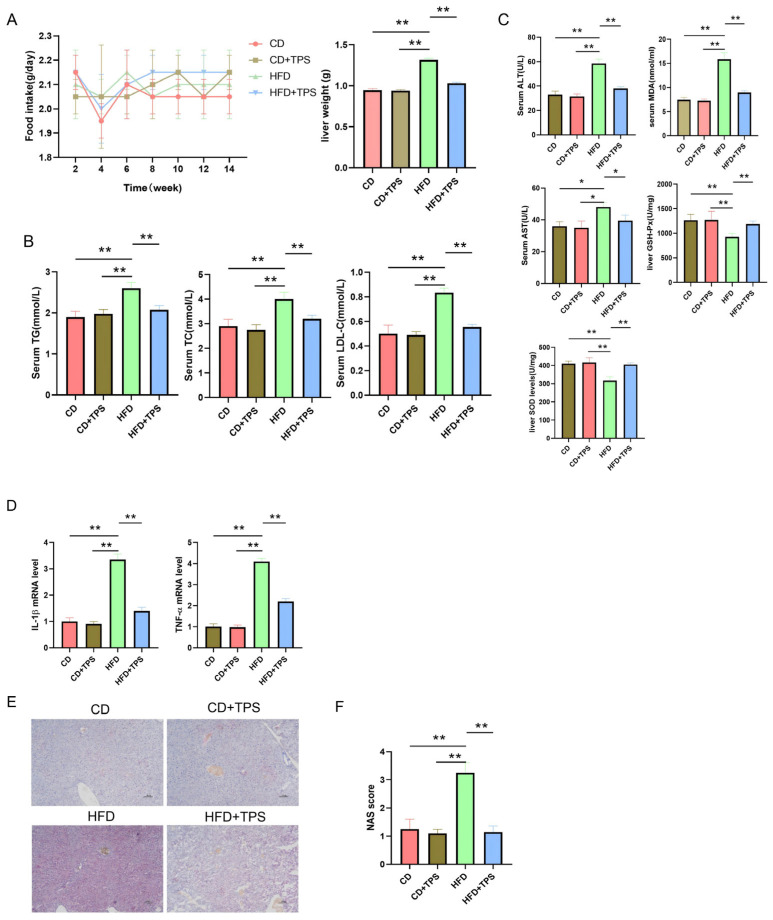
The effect of TPS on NAFLD development. (**A**) The average daily food intake of a mouse per week and liver weight at the end of the experiment. One-way ANOVA with Tukey’s multiple comparisons test: **, *p* < 0.01. (n  =  8/group). (**B**) The level of TG, TC and LDL-C in serum. One-way ANOVA with Tukey’s multiple comparisons test: **, *p* < 0.01. (n = 8/group). (**C**) The level of serum ALT, AST and MDA, and liver GSH-Px and SOD. One-way ANOVA with Tukey’s multiple comparisons test: *, *p* < 0.05; **, *p* < 0.01. (n = 8/group). (**D**) The levels of inflammatory factors (IL-1β and TNF-α) in the liver. One-way ANOVA with Tukey’s multiple comparisons test: **, *p* < 0.01. (n = 8/group). (**E**) Hepatic H&E and Oil red O staining (black scale bar = 100 μm). (**F**) NAS score. One-way ANOVA with Tukey’s multiple comparisons test: **, *p* < 0.01. (n = 8/group). Data are presented as mean ± SD.

To evaluate the preventive effect of TPS on NAFLD, we employed a high-fat diet (HFD)-induced murine model. Throughout the study, daily food intake was monitored, and liver weight was measured upon termination. Although no significant differences in food consumption were observed among the four experimental groups, a marked reduction in liver weight was recorded in TPS-treated mice compared to the HFD controls. For example, liver weight gain in the HFD + TPS group was 50.03% lower than that in the HFD group (*p*-value < 0.01) (Figure 1A). Treatment with TPS also led to substantial improvements in key metabolic parameters. Compared to the HFD controls, the levels of TG in serum decreased by 22.4% (*p* < 0.01), and serum TC and LDL-C were reduced by 15.6% and 25.2%, respectively (*p* < 0.01) (Figure 1B). Furthermore, TPS administration mitigated HFD-induced liver injury, as indicated by decreased serum levels of ALT and AST. As shown in Figure 1C, serum ALT and AST levels in the HFD + TPS group were 42.86% and 28.9% lower than those in the HFD group, respectively (*p* < 0.01). We also detect oxidative stress markers, such as MDA, GSH-Px and SOD, which correlate with NAFLD progression. The serum MDA level in the HFD + TPS group was lower 43.6% than that in the HFD group. Hepatic GSH-Px and SOD activities in the HFD + TPS group were 20.3 and 16.7% than those in the HFD group, respectively (*p* < 0.05).

Meanwhile, TPS also exhibited anti-inflammatory properties, as evidenced by downregulation of the hepatic expression of pro-inflammatory cytokines, including interleukin-1β (IL-1β) and tumor necrosis factor-alpha (TNF-α) (Figure 1D). Oil Red O staining revealed that TPS treatment markedly attenuated hepatic lipid accumulation (Figure 1E). This morphological improvement was further supported by a lower NAFLD activity score (NAS), indicating the efficacy of TPS in suppressing NAFLD progression (Figure 1F). Collectively, these findings suggest that TPS effectively alleviates NAFLD by modulating hepatic lipid metabolism and suppressing inflammatory responses.

### 3.2. Effect of TPS on the Regulation of Macrophage Polarization in NAFLD Model Mice

**Figure 2:**
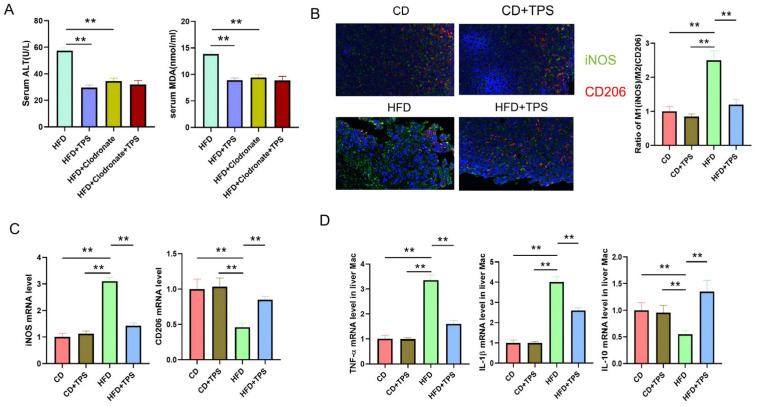
The effect of TPS on liver macrophage polarization in the NAFLD model mice. (**A**) Effect of macrophage on NAFLD development. One-way ANOVA with Tukey’s multiple comparisons test: **, *p* < 0.01. (n = 8/group) (**B**) The expression of iNOS (M1) and CD206 (M2) on liver macrophages of each group. One-way ANOVA with Tukey’s multiple comparisons test: **, *p* < 0.01. (n = 3/group). (**C**) The mRNA expression level of macrophage polarization markers of each group was detected by qRT-PCR. One-way ANOVA with Tukey’s multiple comparisons test: **, *p* < 0.01. (n = 5/group). (**D**) The mRNA expression levels of inflammatory factors in macrophages of each group were detected by qRT-PCR. One-way ANOVA with Tukey’s multiple comparisons test: **, *p* < 0.01. (n = 5/group). Data are presented as mean ± SD.

Previous studies have shown that macrophages play an important role in the pathogenesis of NAFLD [6,7]. Macrophages can differentiate into different polarization types of M1 or M2. The pro-inflammatory cytokines such as TNF-α and IL-1β secreted by M1 macrophages can exacerbate liver damage and promote liver cell apoptosis. M2 macrophages can alleviate the disease progression of NAFLD or NASH [9,10]. First, we conducted a macrophage clearance experiment to verify whether the alleviation of NAFLD and inflammatory response by TPS was related to macrophages. As shown in Figure 2A, clodronate liposomes treatment could delete 90% liver macrophages and resulted in lower serum levels of ALT and MDA being 51.2% and 50.8%, respectively, than those in HFD mice treated without clodronate liposomes (*p* < 0.01), while administration of TPS could not continue to decrease NAFLD development and inflammatory reaction (Figure 2A). This result indicated that clearance of macrophages in HFD mice could partially alleviate NAFLD.

Furthermore, we observed the effect of TPS on liver macrophage polarization. As shown in Figure 2B, compared with the control group, the expression level of iNOS protein, a marker of M1 macrophages, in the liver of HFD group mice was significantly increased, while the expression level of CD206 protein, a marker of M2 macrophages, was significantly decreased. The results suggest that a high-fat diet can induce M1 polarization of mouse liver macrophages. Compared with the HFD group, the TPS treatment group showed a significant decrease in the expression level of iNOS protein and a significant increase in the expression level of CD206 protein in liver macrophages (Figure 2B). The M1/M2 ratio in the liver reduced from 2.5 to 1.3 after TPS treatment. Meanwhile, the mRNA expression levels of macrophage polarization markers detected by qRT-PCR showed a consistent trend with the protein expression levels detected by immunofluorescence. As shown in Figure 2C, the mRNA expression of iNOS in the HFD+TPS group decreased 55% compared with that in the HFD group (*p* < 0.01), whereas the mRNA expression of CD206 increased 45.6% compared with that in the HFD group (*p* < 0.01).

Next, the mRNA expression levels of inflammatory factors in liver macrophages of each group were detected. As shown in Figure 2D, compared with the HFD group, the mRNA expression levels of pro-inflammatory factors such as TNF-α and IL-1β in the liver macrophages of HFD+TPS group mice were significantly decreased by 52.2% (*p* < 0.01), while the level of anti-inflammatory factor IL-10 was significantly increased by almost 2.5-fold (*p* < 0.01). The results suggest that TPS can regulate the macrophage polarization, thereby reducing high-fat diet-induced liver lipid accumulation damage and inflammation.

### 3.3. TPS Affects Macrophage Polarization by Regulating Gut Microbiota Metabolites in NAFLD Mice

**Figure 3:**
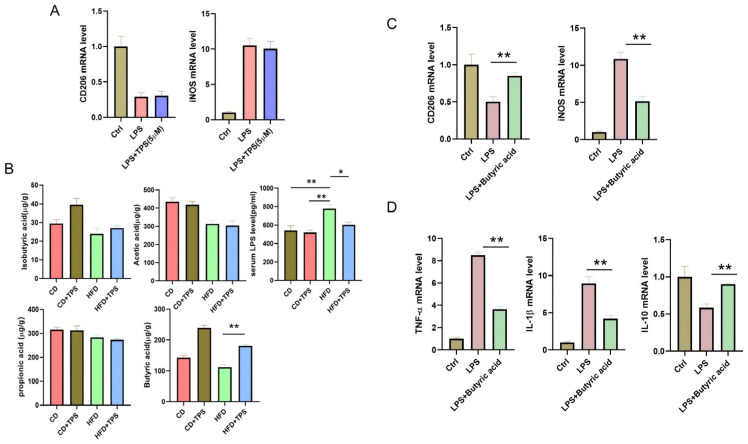
The effect of TPS on the regulation of gut microbiota metabolites in NAFLD model mice. (**A**) Effect of TPS on the expression of iNOS (M1) and CD206 (M2) on liver macrophage in vitro. (**B**) The levels of SCFAs in colon contents and LPS in serum. One-way ANOVA with Tukey’s multiple comparisons test: *, *p* < 0.05; **, *p* < 0.01. (n = 6/group). (**C**) Effect of butyric acid on the expression of iNOS (M1) and CD206 (M2) on liver macrophage in vitro. Unpaired two-tailed *t*-test: **, *p* < 0.01. (n = 3/group). (**D**) Effect of butyric acid on mRNA expression levels of inflammatory factors in macrophages. Unpaired two-tailed *t*-test: **, *p* < 0.01. (n = 3/group). Data are presented as mean ± SD.

To investigate how TPS affects macrophage polarization in the liver, we first treated LPS-stimulated mouse macrophages with TPS in vitro. The results showed that TPS could not directly reverse macrophage polarization from M1 to M2, as evidenced by no changes in expression of M1/M2-related genes (Figure 3A). Previous studies have found that liver macrophage polarization may be influenced by gut microbiota and its metabolites, such as SCFAs and LPS [23,24]. SCFAs are essential in modulating immune responses, influencing the behavior of T cells, B cells, and macrophages, while also curbing pro-inflammatory cytokine production [17,25]. LPS can also promote macrophage polarization towards M1 and induce NAFLD [26,27]. To investigate whether TPS affects macrophage polarization by regulating gut microbiota metabolites, we used gas chromatography to detect the levels of SCFAs in the colon content of each group of mice. Serum LPS levels were detected by ELISA. As shown in Figure 3B, compared with the control group, the levels of serum LPS increased, and SCFAs in colon content were decreased in the HFD group mice, with a particularly significant decrease in butyric acid by 51.2% (*p* < 0.01). TPS treatment could significantly increase butyric acid levels by 83.4% and decrease serum LPS levels by 19.2% compared with the HFD group mice. However, the levels of the other SCFAs were not statistically changed after TPS treatment. This suggests that butyric acid and LPS were essential for liver macrophage polarization regulated by TPS. To further confirm that butyric acid can affect macrophage polarization, liver macrophages were isolated and simultaneously treated with LPS and butyric acid in vitro. The results showed that compared with only the LPS treatment group, butyric acid treatment significantly inhibited macrophage polarization towards M1, as evidenced by a 53.2% decrease in mRNA expression of M1 marker (iNOS) and a 67.5% increase in mRNA expression of M2 marker (CD206) (Figure 3C). Meanwhile, treatment of butyric acid also inhibited the production of pro-inflammatory factors, as evidenced by mRNA expression of TNF-α and IL-1β decreasing by 75% and 62.5%, respectively, and increased expression of anti-inflammatory factors IL-10 by 52.8% (Figure 3D).

### 3.4. TPS Is Prone to Being Broken Down to Butyric Acid by Gut Microbiota

**Figure 4:**
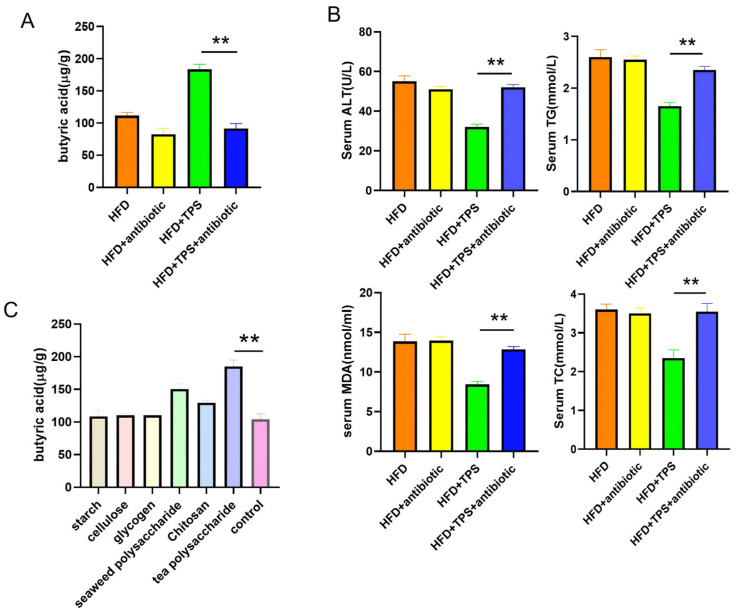
TPS are prone to being broken down to butyric acid by gut microbiota. (**A**) Antibiotics inhibit butyric acid production from TPS. Unpaired two-tailed *t*-test: **, *p* < 0.01. (n = 6/group). (**B**) Antibiotics compromised the therapeutic effect of TPS on NAFLD development. Unpaired two-tailed *t*-test: **, *p* < 0.01. (n = 6/group). (**C**) The level of butyric acid production in the liver from NAFLD model mice fed with various polysaccharides. Unpaired two-tailed *t*-test: **, *p* < 0.01. (n = 6/group). Data are presented as mean ± SD.

Different polysaccharides can be broken down by gut microbiota into different types of short-chain fatty acids. To further observe whether TPS is preferentially broken down into butyric acid by gut microbiota, we fed high-fat diet mice with drinking water containing an antibiotic to deplete the gut microbiota and then administered TPS. The results showed that feeding TPS to high-fat diet mice with antibiotics significantly reduced the content of butyric acid in the colon compared to the group treated without antibiotics (Figure 4A). Antibiotic feeding also significantly inhibited the efficacy of TPS in treating NAFLD, as evidenced by higher liver injury indicators compared to the control group treated without antibiotics (Figure 4B). To observe whether TPS are more easily broken down into butyric acid by the gut microbiota compared to other polysaccharides, we administered different polysaccharides to mice and measured the content of butyric acid in their colon. As shown in Figure 4C, compared to other polysaccharides, feeding TPS produced the highest level of butyric acid in the colon, indicating that TPS are more easily broken down into butyric acid by the gut microbiota.

## 4. Discussion

In our current study, we found that TPS exerted a significant preventive effect on NAFLD and could markedly inhibit the occurrence and development of NAFLD in high-fat diet mice. While previous studies have characterized the lipid-lowering properties of various polysaccharides, our data uniquely highlight the role of the gut–liver axis as a primary mediator of TPS efficacy. We observed that TPS can reduce “intestinal leakage” and inhibit M1 polarization of liver macrophages by promoting the production of butyric acid by the gut microbiota, thereby reducing liver inflammation and preventing pathological changes in NAFLD via the gut–liver axis.

Our study shows that TPS was more likely to promote the production of butyric acid by gut microbiota in HFD mice, which is vital for the inhibition of NAFLD development by TPS. Recent studies have shown that butyric acid is the preferred energy source for colonic epithelial cells. It can promote the synthesis of tight junction proteins such as Occludin and ZO-1, stimulate mucus secretion and enhance the defense function of the intestinal barrier [28]. A robust intestinal barrier means fewer bacterial endotoxins enter the portal circulation through “intestinal leakage”. Endotoxins (LPS) are the main component of intestinal bacteria and play a crucial role in liver inflammation and macrophage polarization in NAFLD [23]. Clinical evidence indicates that in NAFLD patients, impaired colonic mucosal immunity resulting from intestinal dysbiosis allows LPS to translocate from the gut lumen to the liver [24]. On the cytoplasmic membrane of hepatic cells, TLR-4 binds LPS as a ligand, inducing receptor dimerization and triggering downstream signaling cascades. This leads to the production of TNF-α and IL-1-β, which exacerbate liver inflammation and promote fibrosis. Our research results also confirmed that TPS could reduce the level of serum LPS in the liver of HFD mice, suggesting that the intestinal barrier was improved by butyric acid generated by TPS.

A central finding of this study is that TPS-derived butyrate acts as a critical signaling molecule to regulate hepatic macrophage plasticity. We found that butyrate shifts liver macrophage polarization from a pro-inflammatory M1 phenotype toward an anti-inflammatory M2 phenotype. This is highly relevant in the context of human NAFLD, where the M1 shift often precedes overt hepatic inflammation [29,30].

Regarding the mechanism by which butyric acid regulates macrophages, based on previous research reports, we speculate that butyric acid, as an SCFA, may regulate macrophage polarization by inhibiting histone deacetylase and activating G protein-coupled receptors. SCFAs (especially butyric acid) can inhibit the activity of HDACs, leading to an increase in histone acetylation levels. This makes the chromatin structure loose, making it easier to initiate gene transcription. HDAC inhibition selectively downregulates the expression of a series of pro-inflammatory genes [31]. It can inhibit the production of key markers of M1 macrophages, such as iNOS and pro-inflammatory cytokines (IL-6, IL-12, etc.). At the same time, butyric acid can promote the expression of M2-related genes. Additionally, SCFAs may also exert regulatory functions by binding to GPCRs on the macrophages. Different SCFAs have varying affinities for these kinds of receptors. Butyric acid is a high-affinity ligand for GPR109A, while propionic acid and acetic acid have higher affinity for GPR41 and GPR43. When SCFAs bind to these receptors, they trigger a series of downstream signaling pathways, including inhibition of cAMP, activation of MAPK and NF-κB pathways. Upon activation by SCFAs, GPR43 can inhibit the activity of NF-κB, thereby reducing the production of pro-inflammatory cytokines. SCFAs (especially butyric acid and acetic acid) have been shown to inhibit the activation of NLRP3 inflammasomes, thereby reducing the maturation and release of powerful pro-inflammatory cytokines IL-1 β and IL-18, which are key components of M1 macrophage response [32,33].

However, some studies have indicated that the effect of butyrate on macrophage polarization is closely related to its concentration. At low concentrations (<2 mM), butyrate inhibits the release of pro-inflammatory factors from macrophages and induces polarization toward the M2 phenotype. At high concentrations (>10 mM), butyrate exacerbates the inflammatory response, induces macrophage polarization toward the M1 phenotype, and may even trigger apoptosis [34,35]. This phenomenon may be attributed to differences in the binding affinity of butyrate to receptors on the macrophage surface. Varying concentrations of butyrate engage different receptors, leading to distinct downstream signaling pathways. In this study, the concentration of butyrate induced in the colon by TPS was below 2 mM. The concentration of butyrate reaching the liver would be even lower via the gut–liver axis, thereby exerting an anti-inflammatory effect and promoting macrophage polarization toward the M2 phenotype.

Intriguingly, our study also showed that TPS had a superior capacity for butyrate production compared to other polysaccharides. This selectivity likely stems from its unique biochemical architecture. TPS is a complex heteropolysaccharide rich in galacturonic acid, arabinose, and galactose. Unlike simple glycans that are rapidly fermented, the complex branching of TPS requires specialized enzymatic machinery found in butyrogenic taxa such as *Faecalibacterium prausnitzii* [36]. Furthermore, our results support the “cross-feeding” hypothesis, where primary fermenters like *Bifidobacterium* degrade TPS into acetate and lactate, which are subsequently converted into butyrate by secondary fermenters, creating a metabolic environment conducive to hepatic health [37].

The present study has some limitations. It did not conduct a comprehensive big data analysis on the changes in gut microbiota and their metabolites under TPS intervention. So, our exploration of mechanisms by which TPS produces more butyric acid is mainly based on previous research. Furthermore, differences in gut microbiota between humans and mice may also lead to variations in the results. Meanwhile, the TPS used in this study was extracted from black tea, and its monosaccharide composition was not comprehensively analyzed. Future research utilizing fractionated TPS will be essential to identify the most bioactive structural components. In addition, the relatively small sample size of animals in our study may affect the reliability of the statistical significance. Finally, while the link between butyrate and macrophage HDAC inhibition is well-supported by literature, the direct intracellular signaling flux in hepatic macrophages under TPS treatment requires deeper validation through transcriptomic profiling and receptor knockout models. Addressing these gaps will further refine our understanding of polysaccharide-based interventions in metabolic disorders.

## 5. Conclusions

In the present study, we demonstrate that TPS effectively attenuates the progression of NAFLD in a high-fat diet-induced mouse model. Specifically, we identified the gut–liver axis as the primary mediator of this protective effect. TPS supplementation reshapes the gut microbiota profile to favor the production of butyric acid, which subsequently drives the reprogramming of hepatic macrophages from a pro-inflammatory M1 phenotype toward an anti-inflammatory M2 state. This research provides a mechanistic basis for the potential use of TPS as a functional food component in the management of NAFLD.

## Figures and Tables

**Table 1:** Primer list.

Genes Name	Forward	Reverse
TNF-a	CCCTCACACTCAGATCATCTTCT	GCTACGACGTGGGCTACAG
iNOS	ATCTTTGCCACCAAGATGGCCTGG	TTCCTGTGCTGTGCTACAGTTCCG
CD206	TAGATTTTGTGGCTTGGGC	TGGTGTGGTGGGTGTGGT
IL-10	AAGGGTTACTTGGGTTGCC	GCTCTTATTTTCACAGGGGAGA
IL-1b	GCAGGCAGTATCACTCATTGT	AGGCTTTTTTGTTGTTCATCTC
GAPDH	GTGTTCCTACCCCCAATGTGT	ATTGTCATACCAGGAAATGAGCTT

## Data Availability

The raw data supporting the conclusions of this article will be made available by the authors on request.

## Supplementary Materials

The following supporting information can be downloaded at: https://www.mdpi.com/article/10.3390/cimb48030338/s1.

## Abbreviations

The following abbreviations are used in this manuscript:

ALT	alanine aminotransferase
LDL-C	low-density lipoprotein cholesterol
TC	total cholesterol
TG	triglyceride
MDA	malondialdehyde
TPS	tea polysaccharides
NAFLD	Non-alcoholic fatty liver disease
AST	aspartate aminotransferase
GSH-Px	glutathione peroxidase
SOD	superoxide dismutase
