# Supplementary figures and images for: Tea Polysaccharides Ameliorates Non-Alcoholic Fatty Liver Disease in Mice via Regulating Macrophages Polarization by Gut Microbial Metabolites
Source: Curr Issues Mol Biol. 2026 Mar 23;48(3):338. doi: 10.3390/cimb48030338 (PMC13025315; doi:10.3390/cimb48030338)

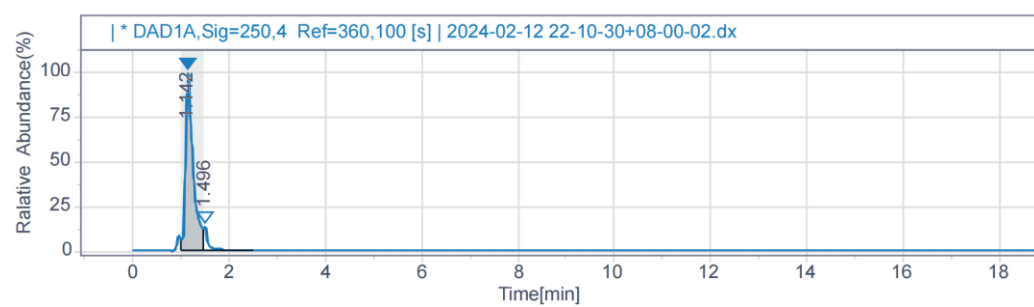

Figure S1 High-performance liquid chromatography profile of TPS

Supplement: Supplementary file 1 [file cimb-48-00338-s001.zip › Figure S1-TPS-HPLC.pdf]
